# Barriers to Creutzfeldt-Jakob Disease Autopsies, California

**DOI:** 10.3201/eid1105.041133

**Published:** 2005-05

**Authors:** Kurt B. Nolte

**Affiliations:** * University of New Mexico, Albuquerque, New Mexico, USA

**Keywords:** pathologist, autopsy, prions, pathology

**To the Editor:** The recent article by Louie et al. underscores a more general disparity between the need for autopsies in potential infectious disease deaths and our present national capacity (1). In addition to confirming Creutzfeldt-Jakob disease (CJD) and allowing the differentiation of classic and variant CJD, autopsies identify previously undetected infections, discover causative organisms in unexplained infectious disease deaths, and provide insights into the pathogenesis of new or unusual infections (2,3). This information is essential for public health and medical interventions.

As outlined by Louie et al., hospital autopsy rates have dropped to single digits, and concerns by pathologists about occupational risks and biosafety have likely contributed to this decline. Currently, the last stronghold of autopsy expertise is forensic pathology (4). However, the medicolegal death investigative system does not have jurisdiction over all potential infectious disease deaths nor is it adequately supported to assume the cases that are missed by our present hospital autopsy system. Additionally, many medicolegal and hospital autopsy facilities with outdated or poorly-designed air flow systems are ill suited to handle autopsies when infectious disease is suspected (5). Air-handling systems can be expensive to fix.

Reference centers such as the National Prion Disease Pathology Surveillance Center, while providing diagnostic expertise, fail to surmount the biosafety obstacles (real and perceived) that prevent pathologists from enthusiastically performing autopsies on those who died of potential infectious diseases, including prion diseases. One potential solution is the creation of regional centers of excellence for infectious disease autopsies that could operate in conjunction with a mobile containment autopsy facility (5,6). Such centers could provide diagnostic expertise as well as biosafety capacity.
